# MedicalAgentsBench for complex medical reasoning: Comparing internalized reasoning models versus externalized agent-based frameworks

**DOI:** 10.1016/j.patter.2026.101610

**Published:** 2026-07-08

**Authors:** Yanjun Shao, Xiangru Tang, Jiwoong Sohn, Jiapeng Chen, Yuxuan Liao, Jiayi Zhang, Jinyu Xiang, Fang Wu, Yilun Zhao, Chenglin Wu, Wenqi Shi, Arman Cohan, Mark Gerstein

**Affiliations:** 1 Program in Computational Biology & Biomedical Informatics, Yale University, New Haven, CT 06520, USA; 2 Department of Computer Science, Yale University, New Haven, CT 06520, USA; 3 Department of Biosystems Science and Engineering, ETH Zurich, 4058 Basel, Switzerland; 4 Department of Computer Science, Stanford University, Stanford, CA 94305, USA; 5 Department of Bioinformatics, UT Southwestern Medical Center, Dallas, TX 75390, USA; 6 Department of Molecular Biophysics and Biochemistry, Yale University, New Haven, CT 06520, USA; 7 Department of Statistics & Data Science, Yale University, New Haven, CT 06520, USA; 8 Department of Biomedical Informatics & Data Science, Yale University, New Haven, CT 06520, USA

**Keywords:** medical reasoning, large language models, multi-agent systems, clinical decision-making, evaluation

## Abstract

Complex medical reasoning requires integrating heterogeneous clinical evidence across multiple inference steps. Large language models (LLMs) now approach this through two routes: internalized reasoning and externalized agent scaffolding (frameworks that decompose problems collaboratively among multiple LLMs). To determine whether these routes are exclusive or complementary, we introduce MedicalAgentsBench, a filtered benchmark of 862 complex clinical questions drawn from the union of eight medical datasets via difficulty-aware curation and contamination screening. Evaluating three internalized reasoning models (DeepSeek-R1, o1-mini, and o3-mini), seven base models, and nine externalized agent-based methods, we find that internalized and externalized approaches each independently improve performance and that their benefits compound: the highest accuracy is achieved by layering agent workflows onto an internalized reasoning model (i.e., o3-mini + MDAgents with 35.1%). Pareto analysis shows this combination dominates the cost-performance frontier; moreover, lightweight optimization on inexpensive models offers an entry point for resource-constrained settings.

## Highlights

• MedicalAgentsBench curates hard medical questions via multi-stage filtering
• Externalized and internalized reasoning are complementary, not competing
• Reasoning models combined with agents dominate the Pareto frontier
• Open-source reasoning models achieve competitive accuracy

## The bigger picture

Hospitals, research labs, and healthcare organizations deploying AI for clinical decision support face a practical question: invest in stronger internalized reasoning models or in multi-agent frameworks that coordinate several AI systems? The question is especially pressing because privacy regulations and infrastructure constraints often require local deployment, limiting access to frontier proprietary models and making the choice between training better local models and wrapping existing ones in agent frameworks a concrete engineering decision. MedicalAgentsBench provides a rigorous evaluation platform that jointly considers accuracy, computational cost, and reasoning depth to inform this choice. Our results show that the approaches are not alternatives but complements; internalizing reasoning (via training-time optimization) improves accuracy while lowering inference cost, and externalizing it (via agent scaffolding) trades additional cost for further accuracy gains. Practitioners may therefore benefit from viewing the two as potentially additive, with the appropriate balance depending on budget and accuracy requirements.

## Teaser

Shao et al. introduce MedicalAgentsBench, a benchmark designed to separate genuine multi-step clinical reasoning from pattern matching. Using this benchmark, they compare internalized reasoning models against externalized agent frameworks and find that the two approaches are complementary; layering agents onto an internalized reasoning model achieves the highest accuracy, exceeding either approach alone.

## Introduction

Clinical reasoning often requires synthesizing heterogeneous evidence (patient history, laboratory values, and imaging findings) across multiple inferential steps to arrive at a diagnosis or management decision. Large language models (LLMs) have demonstrated remarkable capabilities in medical natural language processing, ranging from clinical question answering to diagnostic assistance^1^^,^^2^^,^^3^^,^^4^^,^^5^; yet, as illustrated in Figure 1, even state-of-the-art models (OpenAI o3, GPT-4o, and Claude 3.5 Sonnet) continue to struggle with the multi-step reasoning these scenarios demand.^6^^,^^7^

**Figure 1:**
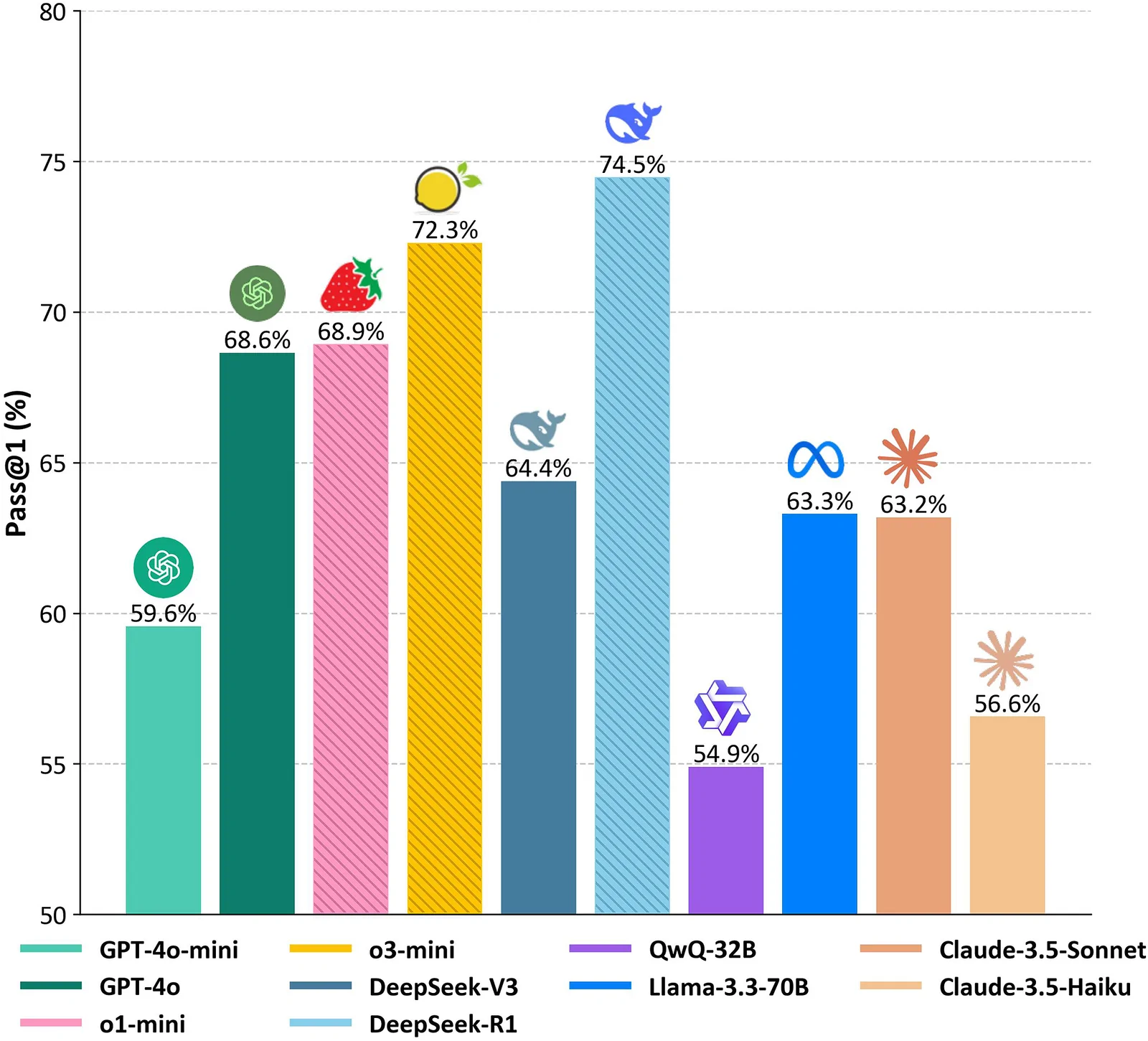
Performance analysis of large language models on medical tasks Overall Pass@1 accuracy comparison across models in zero-shot setting. Hatched bars denote internalized reasoning models. The score is averaged over ten evaluation subsets (MedQA, PubMedQA, MedMCQA, MedBullets, MMLU, MMLU-Pro, AfrimedQA, MedExQA, MedXpertQA-R, and MedXpertQA-U).

The evolution of medical reasoning evaluation has progressed with numerous specialized datasets. Popular benchmarks such as MedQA,^8^ PubMedQA,^9^ and MedMCQA^10^ established the foundation with standardized multiple-choice questions from medical licensing exams and PubMed abstracts. The field then expanded to domain-specific resources such as clinical notes^11^ and question summarization,^12^ integrating visual elements through datasets such as pathology images^13^ and radiology questions.^14^^,^^15^ To address linguistic and regional diversity, multilingual datasets emerged.^16^^,^^17^^,^^18^ The ecosystem further matured with comprehensive collections such as MultiMedQA,^19^ which combines seven distinct medical datasets.

Research in applying LLMs to medical tasks has progressed through several distinct phases. Initial efforts focused on evaluating general-purpose LLMs in medical contexts.^20^^,^^21^^,^^22^ General foundation models^19^^,^^23^ achieved physician-level performance through prompt engineering, followed by domain-specialized open-source models.^24^^,^^25^^,^^26^ The field then advanced toward specialized reasoning methods^7^^,^^27^ and retrieval-augmented generation.^28^^,^^29^^,^^30^ More recently, agent-based frameworks such as MedAgents^31^ and MDAgents^32^ have shown promise by leveraging collaborative multi-agent systems for complex medical decision-making.

To improve LLM reasoning on such tasks, the community has explored a spectrum of approaches that differ in how reasoning is structured and externalized. As summarized in Table 1, existing methods can be broadly categorized into traditional prompting, advanced prompting, discussion-based agent frameworks, and agentic workflow optimization methods, reflecting increasing degrees of reasoning externalization and structural optimization. Traditional prompting techniques, such as chain of thought (CoT) and self-consistency (SC),^33^^,^^34^ aim to elicit intermediate reasoning steps by prompting models to explicitly verbalize multi-step logical inferences before producing a final answer, with SC further improving robustness through aggregating multiple independently sampled reasoning paths. Advanced prompting methods, including domain-specific approaches such as MedPrompt,^35^ extend this paradigm by introducing structured self-reflection, diversity, or multi-round prompting within a single model instance but typically yield only incremental performance gains in complex medical reasoning settings. In contrast, discussion-based agent frameworks such as MedAgents^31^ and MDAgents^32^ externalize reasoning by coordinating multiple specialized agents that engage in explicit discussion, consultation, and cross-verification, decomposing complex medical reasoning into collaborative subtasks such as hypothesis generation and evidence evaluation. Beyond discussion-based agent frameworks, agentic workflow optimization methods^36^^,^^37^ further automate the design of agent interaction structures by algorithmically optimizing reasoning workflows and control strategies rather than relying on fixed, hand-designed agent protocols. While agent-based approaches can substantially enhance reasoning performance by externalizing deliberation and verification beyond a single model forward pass, they also introduce increased system complexity and computational overhead.

**Table 1:** Methods overview

Method	Description
**Traditional prompting**	

Chain of thought^33^	prompts explicit intermediate reasoning steps before generating a final answer
Self-consistency^34^	aggregates multiple independently sampled reasoning chains via majority voting

**Advanced prompting**	

Multi-Persona^38^	encourages diverse reasoning by assigning distinct personas within a single model
Self-Refine^39^	iteratively refines outputs using model-generated self-feedback

**Discussion-based agent frameworks**	

MedPrompt^35^	uses structured multi-round prompting with ensemble-based answer aggregation
MedAgents^31^	coordinates multiple specialized agents for collaborative medical reasoning
MDAgents^32^	dynamically adapts multi-agent interactions based on task complexity

**Agentic workflow optimization methods**	

AFlow^36^	searches over agentic workflows to optimize reasoning strategies
SPO^37^	performs self-supervised prompt optimization using task-level feedback

The reasoning approaches span four categories: traditional prompting, advanced prompting, discussion-based agent frameworks, and agentic workflow optimization methods.

In parallel, a new class of reasoning models has emerged, including OpenAI o3-mini and DeepSeek R1.^40^ In this work, we use the term “reasoning models” to refer to LLMs whose parameters are explicitly optimized, via reinforcement learning, CoT supervision, or distillation, to perform multi-step reasoning internally rather than relying on external orchestration or tool-based decomposition. A useful overview of this emerging category is provided by Raschka.^41^ In effect, these models amortize reasoning into their parameters: training-time optimization shifts more of the cognitive work upstream, so complex inference can be executed within a single model call at deployment time.^40^ Unlike agent frameworks, these models aim to perform complex reasoning within a single forward pass. This distinction raises a fundamental and unresolved question: in medical reasoning tasks, do internalized reasoning and externalized agent workflows represent exclusive, competing paradigms, or are they complementary approaches that can readily be combined?

Answering this question is hindered by several limitations of existing medical benchmarks. First, as shown in Table 2, current datasets contain a large proportion of straightforward examination-style questions. On such questions, even base LLMs achieve high accuracy (see Table 3, “full” columns), masking meaningful differences between internalized reasoning models and agent frameworks. Second, inconsistent and subjective sampling practices across prior studies^31^^,^^32^ prevent reliable cross-method comparison, particularly between approaches that differ substantially in cost and inference structure. Third, existing benchmarks rarely evaluate performance jointly with computational cost and inference time, despite these factors being central to the design trade-offs in agent-based systems.

**Table 2:** Medical question-answering datasets

Benchmark	Size	Average length	Options	Description
**Professional medical exam benchmarks**				

MedQA^8^	1,273	167.1	4	medical licensing exam multiple-choice questions
MedMCQA^10^	2,816	18.7	4	AIIMS and NEET PG medical entrance exam questions

**Biomedical literature and educational QA benchmarks**				

PubMedQA^9^	500	316.1	3	biomedical research questions based on PubMed abstracts
MedBullets^42^	308	213.1	5	educational medical questions from the MedBullets platform
MedExQA^43^	935	19.1	4	multi-specialty medical questions with expert explanations
MedXpertQA^44^	2,450	257.4	10	expert-level medical questions for understanding and reasoning

**General-domain benchmarks with medical coverage**				

MMLU^45^	1,089	55.9	4	multitask academic benchmark including medical subjects
MMLU-Pro^46^	818	57.4	3–10	harder MMLU variant emphasizing deeper reasoning
MedicalAgentsBench	862	147.4	3–10	high-difficulty medical reasoning subset across benchmarks

Benchmarks cover professional medical examinations, biomedical literature and education, and general-domain reasoning tasks with medical content.

**Table 3:** Performance by model and task

Model	MedQA	PubMedQA	MedMCQA	MedBullets	MMLU	MMLU-Pro	MedExQA	MedXpert-R	MedXpert-U
**Full test set**									

GPT-4o-mini	73.2 ± 1.2	76.6 ± 2.0	65.7 ± 0.8	53.9 ± 0.6	84.3 ± 1.7	57.1 ± 0.5	78.8 ± 2.2	13.7 ± 0.9	16.9 ± 1.1
GPT-4o	88.2 ± 1.4	**79.3** ± **1.7**	76.3 ± 1.1	70.6 ± 1.4	90.9 ± 0.9	68.6 ± 1.7	84.8 ± 1.7	22.6 ± 0.6	23.5 ± 2.4
DeepSeek-V3	79.1 ± 1.1	73.7 ± 1.9	74.4 ± 0.7	60.6 ± 0.6	89.5 ± 1.0	65.1 ± 1.1	83.8 ± 1.6	19.0 ± 2.4	23.3 ± 2.4
o1-mini	89.6 ± 1.7	77.2 ± 0.7	73.3 ± 0.6	73.5 ± 1.3	91.1 ± 2.2	67.4 ± 1.1	82.4 ± 0.8	29.5 ± 0.6	26.8 ± 2.0
o3-mini	**93.0** ± **0.5**	79.3 ± 1.9^a^	77.5 ± 2.0^a^	**82.2** ± **1.2**	93.5 ± 2.2^a^	69.7 ± 1.2^a^	85.6 ± 1.1^a^	34.0 ± 2.0^a^	31.8 ± 2.3^a^
QwQ-32B	78.9 ± 0.7	78.2 ± 2.0	69.8 ± 2.0	54.5 ± 1.5	87.2 ± 0.6	65.5 ± 0.6	81.4 ± 1.1	17.8 ± 2.3	17.3 ± 1.3
DeepSeek-R1	91.8 ± 1.0^a^	75.7 ± 1.1	**82.2** ± **2.4**	78.8 ± 1.8^a^	**95.3** ± **2.1**	**79.7** ± **2.3**	**86.2** ± **2.1**	**37.4** ± **1.1**	**37.7** ± **1.0**
Llama-3.3-70B	76.8 ± 2.1	78.1 ± 0.5	71.3 ± 1.3	61.2 ± 0.7	85.6 ± 2.4	61.6 ± 1.5	78.9 ± 1.2	20.3 ± 2.4	22.6 ± 1.5
Claude-3.5-Sonnet	78.1 ± 1.1	76.5 ± 1.7	68.7 ± 0.6	56.3 ± 2.3	86.5 ± 0.8	63.7 ± 2.5	81.3 ± 1.8	20.3 ± 1.0	24.1 ± 1.2
Claude-3.5-Haiku	63.1 ± 1.8	73.7 ± 0.7	63.0 ± 1.1	49.2 ± 0.6	79.7 ± 1.9	57.6 ± 1.5	76.9 ± 1.8	14.1 ± 1.9	16.9 ± 2.4

**Hard test set**									

GPT-4o-mini	21.8 ± 2.4	10.2 ± 1.7	17.1 ± 0.8	10.1 ± 2.2	12.2 ± 1.9	11.2 ± 2.4	4.1 ± 0.9	5.9 ± 0.9	5.2 ± 1.5
GPT-4o	32.2 ± 1.1	8.8 ± 0.8	25.2 ± 1.2^a^	18.8 ± 2.1	24.8 ± 1.5	20.8 ± 0.6	18.1 ± 0.8	6.8 ± 2.4	5.8 ± 2.1
DeepSeek-V3	16.1 ± 0.7	12.2 ± 1.4	19.0 ± 1.5	13.4 ± 2.3	14.9 ± 1.8	11.9 ± 1.5	7.1 ± 0.9	6.0 ± 2.1	9.0 ± 2.3
o1-mini	49.1 ± 2.3^a^	10.7 ± 0.9	20.8 ± 1.2	38.5 ± 1.0	31.4 ± 1.2	19.3 ± 1.6	15.3 ± 2.1	**29.2** ± **2.5**	13.9 ± 0.9
o3-mini	**52.9** ± **2.1**	16.0 ± 2.0^a^	24.1 ± 0.6	**50.3** ± **0.7**	35.9 ± 1.7^a^	15.0 ± 0.6	18.1 ± 1.2^a^	25.1 ± 1.8^a^	14.9 ± 1.4^a^
QwQ-32B	29.2 ± 1.9	**16.2** ± **1.6**	24.0 ± 1.5	12.4 ± 1.4	19.4 ± 0.7	27.9 ± 1.8^a^	9.8 ± 1.5	8.7 ± 1.0	6.0 ± 2.0
DeepSeek-R1	47.1 ± 0.7	12.7 ± 0.8	**30.9** ± **2.1**	43.8 ± 2.2^a^	**43.7** ± **0.9**	**36.8** ± **1.6**	**26.0** ± **2.3**	25.1 ± 0.7	**26.3** ± **1.4**
Llama-3.3-70B	13.9 ± 2.2	12.9 ± 1.5	19.7 ± 0.9	17.1 ± 1.2	12.4 ± 1.1	9.8 ± 1.9	6.9 ± 2.4	9.1 ± 1.0	8.9 ± 1.1
Claude-3.5-Sonnet	18.1 ± 0.6	10.1 ± 1.5	9.8 ± 1.1	9.1 ± 1.0	16.2 ± 1.5	13.7 ± 1.0	13.1 ± 2.0	8.8 ± 2.0	11.0 ± 1.8
Claude-3.5-Haiku	13.0 ± 1.6	12.1 ± 2.2	22.7 ± 0.9	10.2 ± 1.7	11.2 ± 0.5	11.8 ± 1.0	13.1 ± 0.8	8.1 ± 1.3	6.0 ± 0.8

Accuracy values are in percentages with standard errors, reported as mean ± SD across 3 runs. The best values are highlighted in bold.

a Second-best values.

To address these challenges, we introduce MedicalAgentsBench, a benchmark specifically designed to enable a principled comparison between internalized reasoning models and externalized agent frameworks for complex medical reasoning tasks. Unlike existing benchmarks that either focus on general medical knowledge or suffer from ceiling effects, MedicalAgentsBench employs a pipeline that (1) draws from eight diverse established medical datasets (MedQA, PubMedQA, MedMCQA, MedBullets, Massive Multitask Language Understanding (MMLU), MMLU-Pro, MedExQA, and MedXpertQA), (2) applies difficulty-based filtering to select questions that challenge current models (specifically, questions where fewer than 50% of tested models provide correct answers), (3) conducts thorough contamination analysis to ensure validity, and (4) incorporates human annotations from medical professionals to verify reasoning depth requirements. Our comprehensive experiments yield several key insights: (1) both externalized agent scaffolding and internalized reasoning independently improve performance, and their benefits are complementary: layering agent workflows on internalized reasoning models (e.g., o3-mini + MDAgents) yields the highest overall accuracy; (2) the Pareto frontier of cost-performance trade-offs is dominated by reasoning model-agent combinations at the high end and by lightweight workflow optimization methods (e.g., SPO) at the low end; and (3) open-source reasoning models achieve competitive accuracy, though their extended reasoning chains do not necessarily reduce per-sample inference cost.

Concurrent with our work, several related evaluation efforts have emerged. MedHELM^47^ provides a holistic evaluation framework for LLMs across a broad range of medical tasks. MedAgentBench^48^ introduces a virtual electronic health record (EHR) environment for benchmarking clinical agent capabilities. MedAgentBoard^49^ benchmarks multi-agent collaboration against conventional methods across diverse medical tasks. MedAgentGym^50^ proposes a scalable agentic training environment for code-centric reasoning in biomedical data science. Together, these works reflect the growing community interest in rigorous, principled evaluation of LLMs and agent-based systems in medical contexts.

## Results

### Benchmark overview

MedicalAgentsBench is a carefully curated benchmark designed to evaluate complex medical reasoning tasks. Drawing from eight established medical datasets (MedQA,^8^ PubMedQA,^9^ MedMCQA,^10^ MedBullets,^42^ MMLU,^45^ MMLU-Pro,^46^ MedExQA,^43^ and MedXpertQA^44^), we systematically construct a challenging subset of 862 questions (average token length: 147.4) that focuses on complex reasoning scenarios. Our multi-stage construction pipeline combines difficulty-aware curation (selecting questions where fewer than 50% of tested models answer correctly), contamination screening via memorization effects Levenshtein detector (MELD),^51^ and human validation of reasoning depth by four medical professionals using a five-level rubric (see methods for full details). As shown in Table 3, while models achieve high accuracy on the full set (e.g., GPT-4o: 88.2% on MedQA), their performance drops significantly on our hard subset (e.g., GPT-4o: 32.2%), confirming that the selected questions demand deeper analytical processing and multi-step medical reasoning.

### Quantitative evaluations

Most models struggle with our challenging benchmark, with even powerful systems such as GPT-4o achieving only 32.2% accuracy on MedQA and 18.1% on MedExQA in our hard subset. This substantial drop in performance confirms that our selection criteria effectively identify questions that require advanced reasoning. Amid these challenges, DeepSeek-R1 demonstrates remarkable performance, achieving the highest scores on five datasets (MedMCQA: 30.9%, MMLU: 43.7%, MMLU-Pro: 36.8%, MedExQA: 26.0%, and MedXpertQA-U: 26.3%). Similarly, o3-mini excels on two datasets: MedQA (52.9%) and MedBullets (50.3%).

The central finding from our method comparison (Table 4) is that externalized and internalized reasoning are complementary. Agent frameworks substantially improve base models: for instance, GPT-4o + AFlow (29.8% average) outperforms bare GPT-4o zero-shot by a wide margin. Reasoning models independently advance the frontier: bare o3-mini (27.8%) already surpasses most agent-augmented base-model configurations. Critically, layering agent workflows on top of reasoning models yields further gains: o3-mini + MDAgents achieves the highest overall accuracy (35.1%), followed by o3-mini + Multi-Persona (34.1%) and o3-mini + CoT (32.7%), all surpassing bare o3-mini by 5–7 percentage points. This indicates that agents add value even when the underlying model is already strong, most likely by introducing diverse reasoning paths and catching errors that a single model pass would miss.

**Table 4:** Performance by task and method

Method	MedQA	PubMedQA	MedMCQA	MedBullets	MMLU	MMLU-Pro	MedExQA	MedXpert-R	MedXpert-U	Average
**GPT-4o-mini**										

CoT	24.7 ± 3.2	14.3 ± 1.5	23.3 ± 2.5	17.6 ± 0.6	26.0 ± 2.4	35.7 ± 2.1^a^	15.7 ± 1.5	7.3 ± 2.3	12.3 ± 2.5	19.7 ± 0.5
CoT-SC	21.7 ± 2.1	13.3 ± 2.1	22.3 ± 2.1	15.7 ± 1.1	27.9 ± 0.8^a^	35.7 ± 1.5^a^	**16.3** ± **3.1**	7.7 ± 2.1	**14.0** ± **1.0**	19.4 ± 0.3
Multi-Persona	30.3 ± 1.5	16.3 ± 4.2	21.7 ± 0.6	16.1 ± 1.7	26.9 ± 1.6	35.0 ± 1.0	15.7 ± 2.3	6.7 ± 1.5	11.0 ± 1.0	20.0 ± 0.6
Self-Refine	31.0 ± 3.6^a^	13.3 ± 3.2	24.3 ± 0.6^a^	17.6 ± 1.7	**29.7** ± **2.1**	32.0 ± 2.6	16.0 ± 3.0^a^	9.0 ± 2.0	**14.0** ± **1.7**	**20.8** ± **0.9**
MedPrompt	21.0 ± 3.5	13.7 ± 3.2	18.3 ± 1.5	18.4 ± 1.3	22.8 ± 2.1	**36.0** ± **3.0**	14.3 ± 1.2	7.3 ± 1.5	10.7 ± 1.5	18.1 ± 0.5
MedAgents	25.3 ± 2.3	12.3 ± 1.5	22.0 ± 1.0	18.7 ± 2.8^a^	24.2 ± 3.4	31.3 ± 2.5	12.0 ± 0.0	7.7 ± 2.1	10.7 ± 1.5	18.3 ± 0.4
MDAgents	24.0 ± 1.0	17.0 ± 5.6	22.0 ± 1.3	14.2 ± 1.7	20.5 ± 2.4	32.0 ± 2.0	12.3 ± 1.5	5.3 ± 1.2	7.0 ± 2.0	17.2 ± 1.1
SPO	17.7 ± 1.5	**25.7** ± **2.1**	20.7 ± 0.6	**22.5** ± **0.0**	22.4 ± 2.9	32.0 ± 1.0	15.0 ± 1.0	**11.7** ± **1.2**	13.3 ± 2.1^a^	20.1 ± 0.7
AFlow	**32.3** ± **2.1**	18.7 ± 3.5^a^	**28.0** ± **3.0**	16.9 ± 1.1	25.6 ± 1.6	28.7 ± 1.5	10.0 ± 3.0	10.7 ± 3.5^a^	10.3 ± 3.5	20.1 ± 2.1^a^

**GPT-4o**										

CoT	44.0 ± 4.4	11.3 ± 3.2	31.3 ± 1.5^a^	30.7 ± 2.3	31.1 ± 4.4	37.7 ± 2.3	21.0 ± 2.6	14.3 ± 2.1	14.0 ± 1.0	26.2 ± 1.6
CoT-SC	42.3 ± 5.0	7.3 ± 1.5	**32.7** ± **2.5**	31.1 ± 0.6	31.5 ± 1.4	**39.7** ± **4.2**	20.7 ± 2.3	14.0 ± 4.6	13.7 ± 1.5	25.9 ± 0.7
Multi-Persona	45.0 ± 1.0	17.3 ± 2.1	29.7 ± 4.2	34.5 ± 4.5^a^	34.7 ± 2.9	39.3 ± 2.5^a^	21.0 ± 2.0	16.7 ± 5.8^a^	13.0 ± 2.6	27.9 ± 1.3^a^
Self-Refine	45.3 ± 3.8^a^	15.0 ± 2.0	29.3 ± 4.0	33.3 ± 5.1	36.1 ± 2.1^a^	33.3 ± 3.1	20.3 ± 1.5	16.0 ± 1.0	16.7 ± 2.1	27.3 ± 0.3
MedPrompt	37.3 ± 1.5	11.0 ± 3.0	28.0 ± 1.0	24.7 ± 3.0	32.4 ± 0.8	36.3 ± 3.5	21.0 ± 1.0	14.3 ± 1.5	13.3 ± 0.6	24.3 ± 0.3
MedAgents	43.0 ± 1.0	17.7 ± 2.5	30.3 ± 0.6	29.6 ± 2.3	29.2 ± 4.8	37.7 ± 2.5	20.3 ± 1.5	13.0 ± 1.7	12.3 ± 2.1	25.9 ± 1.1
MDAgents	37.3 ± 1.5	20.7 ± 2.5	27.2 ± 1.1	19.9 ± 5.5	28.8 ± 3.6	**39.7** ± **1.5**	21.3 ± 1.2^a^	10.9 ± 2.5	11.9 ± 1.4	24.2 ± 0.8
SPO	32.0 ± 1.7	**31.3** ± **1.5**	30.0 ± 2.0	28.8 ± 0.6	34.7 ± 2.1	36.7 ± 0.6	21.0 ± 2.0	**17.0** ± **2.0**	17.3 ± 1.5^a^	27.7 ± 0.8
AFlow	**47.0** ± **1.0**	23.3 ± 4.6^a^	31.3 ± 1.5^a^	**36.0** ± **1.9**	**37.9** ± **3.4**	36.0 ± 3.6	**23.0** ± **2.6**	14.3 ± 2.3	**19.7** ± **1.5**	**29.8** ± **1.0**

**o1-mini**										

CoT	48.0 ± 4.4	13.7 ± 2.1	24.7 ± 2.3	**43.8** ± **0.0**	32.4 ± 3.2	29.0 ± 4.0	15.0 ± 3.0	29.3 ± 4.0^a^	**18.3** ± **2.1**	28.2 ± 0.9
CoT-SC	49.7 ± 2.3	15.0 ± 2.0	24.7 ± 2.5	40.4 ± 1.9	30.6 ± 0.8	17.3 ± 1.5	16.3 ± 1.2	26.3 ± 1.2	16.0 ± 2.6	26.3 ± 0.6
Multi-Persona	50.0 ± 2.6^a^	19.3 ± 2.3^a^	24.7 ± 2.3	42.7 ± 2.2^a^	33.8 ± 0.8^a^	30.0 ± 1.0	17.3 ± 2.3	**31.0** ± **5.3**	16.0 ± 4.4	**29.4** ± **1.5**
Self-Refine	**50.7** ± **7.4**	**20.0** ± **5.0**	**27.3** ± **3.2**	39.7 ± 4.7	**33.8** ± **3.4**	30.0 ± 5.3	16.0 ± 1.7	28.7 ± 2.5	17.0 ± 1.7^a^	29.2 ± 1.1^a^
MedPrompt	42.3 ± 3.1	15.7 ± 1.5	21.0 ± 3.0	38.6 ± 1.3	28.3 ± 2.9	33.0 ± 4.0^a^	18.7 ± 1.5^a^	26.7 ± 1.5	16.3 ± 2.5	26.7 ± 0.5
MedAgents	47.0 ± 4.4	18.0 ± 2.0	25.7 ± 2.9^a^	37.1 ± 1.1	27.4 ± 6.0	**34.7** ± **1.5**	14.0 ± 3.0	24.3 ± 2.5	13.3 ± 2.5	26.8 ± 1.4
MDAgents	39.4 ± 3.4	18.3 ± 1.0	24.7 ± 0.6	36.9 ± 2.4	28.0 ± 2.9	32.9 ± 0.5	**18.7** ± **1.6**	21.2 ± 2.6	13.6 ± 2.3	26.0 ± 0.7

**o3-mini**										

CoT	56.7 ± 2.1	15.0 ± 1.7	27.7 ± 3.2^a^	49.1 ± 1.3	38.4 ± 2.7	40.3 ± 2.1^a^	19.7 ± 1.5	28.0 ± 3.6	22.7 ± 3.2	33.0 ± 0.9
CoT-SC	55.0 ± 1.7	14.7 ± 1.5	26.0 ± 1.0	50.2 ± 2.3	37.9 ± 0.8	38.3 ± 0.6	18.7 ± 1.2	28.0 ± 2.0	22.3 ± 2.1	32.3 ± 0.5
Multi-Persona	**61.0** ± **1.0**	16.7 ± 2.9	25.7 ± 2.1	54.7 ± 0.6^a^	39.3 ± 2.9^a^	40.3 ± 2.1^a^	20.3 ± 3.1^a^	29.7 ± 2.9^a^	22.7 ± 1.5	34.5 ± 0.7^a^
Self-Refine	52.7 ± 2.1	17.3 ± 1.5^a^	27.7 ± 2.5^a^	50.9 ± 1.7	36.5 ± 0.8	31.7 ± 1.5	**22.0** ± **2.0**	**32.8** ± **1.4**	22.3 ± 1.2	32.7 ± 0.7
MedPrompt	59.7 ± 1.5^a^	15.3 ± 1.2	27.3 ± 1.5	**57.3** ± **4.5**	33.8 ± 2.1	37.3 ± 0.6	18.3 ± 1.5	29.7 ± 0.6	23.3 ± 3.1^a^	33.6 ± 0.6
MedAgents	58.0 ± 4.0	16.3 ± 0.6	26.3 ± 2.5	53.5 ± 0.7	34.7 ± 1.6	38.0 ± 4.0	13.0 ± 0.0	28.7 ± 2.5	23.0 ± 2.6	32.4 ± 1.4
MDAgents	58.5 ± 2.3	**20.4** ± **4.6**	**29.9** ± **2.2**	54.4 ± 1.5	**41.1** ± **6.3**	**41.3** ± **5.0**	18.3 ± 1.5	29.6 ± 0.9	**23.5** ± **2.5**	**35.2** ± **1.1**

All tasks are evaluated on the hard set. Each model is evaluated separately with all applicable methods. Accuracy values are in percentages, reported as mean ± SD over 3 runs. The best values are highlighted in bold.

a Second-best values.

Among individual methods, automated workflow optimization (AFlow) and multi-persona prompting (Multi-Persona) achieve the strongest results when applied to base models, while discussion-based agent frameworks (MedAgents and MDAgents) show their greatest value when paired with reasoning models. Traditional prompting gains from CoT-SC over CoT are inconsistent on this hard subset, and domain-specific methods such as MedPrompt lack consistency across datasets (see Table 4 for full per-dataset results). Additional qualitative error analysis and representative failure cases are provided in Note S1.

### Benchmark quality assessment

We assess benchmark quality along two dimensions: reasoning depth to verify cognitive difficulty and data contamination to ensure fair and reliable model comparison.

#### Reasoning depth analysis

To directly assess whether MedicalAgentsBench emphasizes multi-step clinical reasoning rather than superficial difficulty or obscure knowledge retrieval, we conducted an expert-annotated analysis of reasoning depth using a standardized five-level rubric, as detailed in the human validation protocol in the methods. Four medical students in their clinical years independently annotated a stratified sample of 20 questions per dataset (10 from the full split and 10 from the hard split), covering all eight source datasets across nine evaluation subsets (180 questions total). This design enables a direct comparison of the cognitive demands imposed by the original datasets versus the curated benchmark. Figure 2 reveals a clear structural shift in cognitive demands between the two splits. In the full distributions of generalist benchmarks such as MMLU and MMLU-Pro, reasoning depth is bottom heavy, with a predominance of level 1 and 2 questions solvable via direct fact retrieval or single-step deduction. In contrast, the hard subset exhibits a pronounced shift toward levels 4 and 5, corresponding to multi-hop clinical reasoning that requires synthesizing heterogeneous evidence or resolving competing hypotheses.

**Figure 2:**
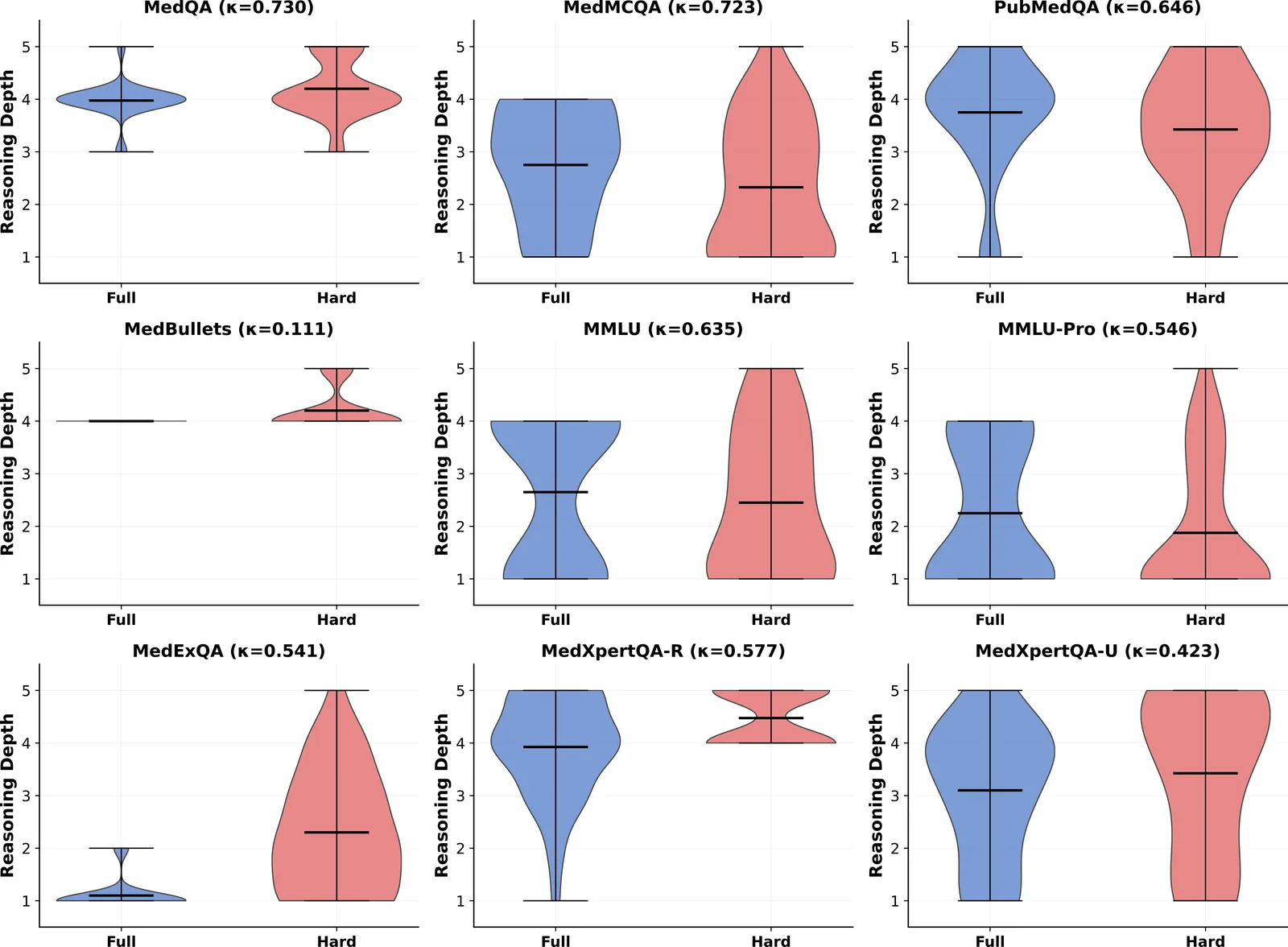
Reasoning depth distribution by dataset split Violin plots comparing the density of reasoning depth ratings (levels 1–5) between the complete source datasets (full) and the curated MedicalAgentsBench subset (hard). *κ* values indicate inter-annotator agreement for each domain. Full details of the annotation can be found in the human validation protocol in the methods.

This pattern is consistent across diverse medical domains and is not driven by a small number of outlier datasets. For example, in MedXpertQA, the hard subset effectively removes direct-recall questions and concentrates on scenarios that require integrating patient history, vital signs, and diagnostic findings to arrive at a diagnosis or management decision. Even for case-based datasets such as MedBullets, which already operate at relatively high reasoning levels, the hard split further emphasizes level 5 reasoning involving conflicting clinical evidence or subtle contraindications. Inter-annotator agreement is substantial (overall Fleiss’ *κ* = 0.62; Table S3), indicating that these distinctions in reasoning depth are clinically meaningful and reproducible rather than subjective.

#### Data contamination analysis

We assessed potential data contamination using the MELD framework.^51^ MELD splits each question into two halves: the first half is provided as context, and the model is prompted to generate the second half. The Levenshtein distance ratio between the generated output and the original unseen portion quantifies memorization; higher similarity indicates that the model has likely encountered the question during training. This approach is particularly well suited to multiple-choice benchmarks, where verbatim reproduction of question text, rather than answer choices, serves as the primary indicator of training exposure (Figure 3). MELD has high precision but unknown recall: a detected match strongly indicates memorization, but absence of a match does not guarantee clean data.

**Figure 3:**
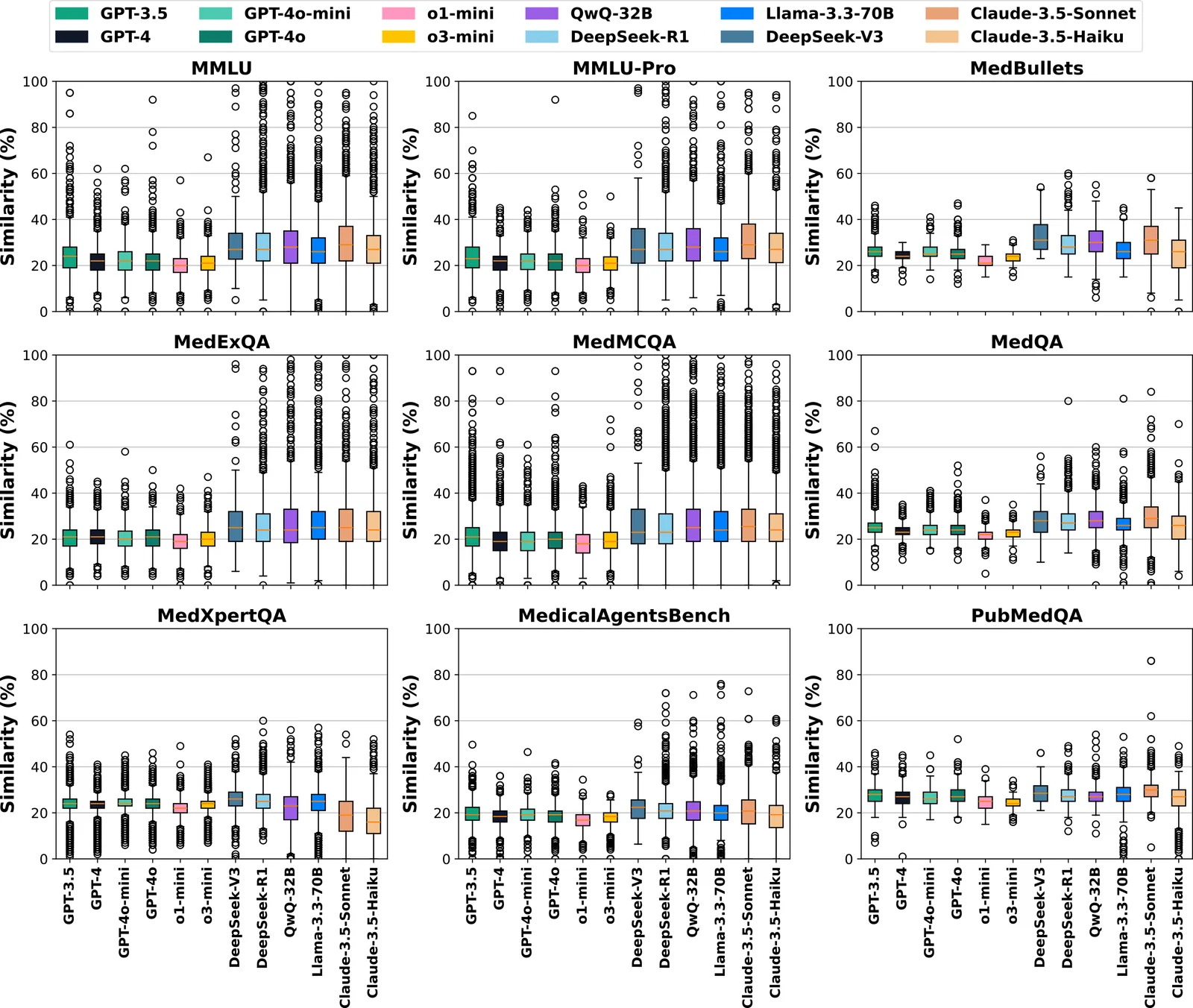
Data contamination analysis across medical question-answering datasets using MELD The boxplots display similarity percentages between model-generated text and original question text, with higher values potentially indicating memorization of training data. Lower similarity scores suggest minimal data contamination, while higher values may indicate potential contamination in model training data.

The analysis reveals a clear divide between commercial and open-source models. OpenAI models show consistently low similarity scores (median: 20%–25%), suggesting minimal verbatim memorization. In contrast, several open-source models (DeepSeek-R1, QwQ-32B, and Llama-3.3-70B) exhibit substantially higher scores, with some reproducing question texts at over 95% character-level accuracy on MMLU and MMLU-Pro (60%–90% similarity range). Importantly, our hard subset demonstrates significantly lower MELD scores across all models than the corresponding full sets, confirming its utility for more reliable cross-model comparison. A manual review of high-similarity cases in MedMCQA and MedExQA attributed some of the elevated scores to shared standard medical terminology rather than direct data leakage. Given these contamination concerns, our primary method comparisons (Table 4) focus on OpenAI models (GPT-4o-mini, GPT-4o, o1-mini, and o3-mini), which exhibit the lowest contamination risk and therefore provide the most reliable basis for comparing reasoning strategies. Search-based optimization methods (SPO and AFlow) were evaluated only for GPT-4o-mini and GPT-4o.

### Cost-performance analysis

Our evaluation examines both model architecture efficiency and reasoning method effectiveness through a cost-performance lens. We analyze these dimensions separately using two complementary visualizations.

#### Base-model cost efficiency

As shown in Figure 4, the ten base models occupy markedly different positions in accuracy-cost space across the eight datasets. The marker encoding and Pareto frontier are described in the figure caption; here, we focus on the substantive pattern. In each image, the red dashed frontier identifies non-dominated models that achieve the best accuracy attainable at a given inference cost, whereas points below the frontier are strictly inefficient relative to another model on the same dataset. DeepSeek-R1 and o3-mini recur on or near this frontier across many datasets, indicating that they frequently define the strongest empirical trade-offs among the base models.

**Figure 4:**
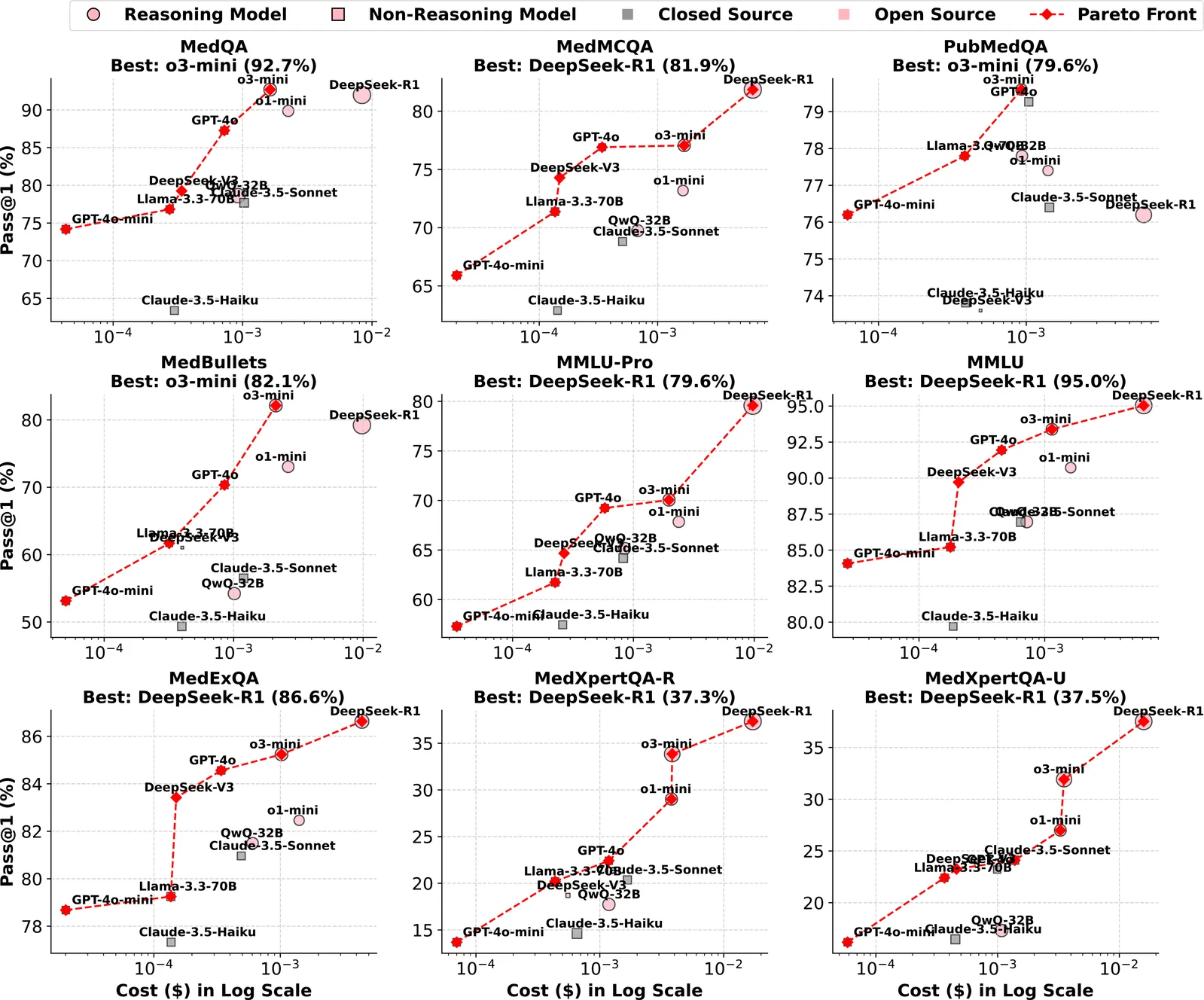
Cost-performance analysis across medical datasets, comparing open- and closed-source language models Each subplot shows the ten base models for one dataset, plotting Pass@1 accuracy (%) against inference cost per sample (USD, log scale). Circles denote internalized reasoning models and squares denote non-reasoning models; pink markers indicate open-source models, and gray markers indicate closed-source models. The red dashed line traces the empirical Pareto frontier, and each subplot title reports the highest-accuracy model for that dataset.

The frontier nevertheless varies by task. MedQA and MedBullets show steep gains as inference cost increases, whereas PubMedQA exhibits a flatter profile with more modest returns to expensive models. Across several of the more challenging datasets, internalized reasoning models occupy stronger positions than non-reasoning models at comparable cost levels, suggesting that explicit training for multi-step reasoning can improve deployment-time efficiency and accuracy. Open-source models demonstrate competitive performance. On the full test set, DeepSeek-R1 achieves the highest accuracy among all models on several benchmarks, including MedMCQA (82.2%) and MMLU-Pro (79.7%), rivaling or surpassing closed-source alternatives. However, its extended reasoning chains result in higher per-sample inference costs than compact closed-source models such as o3-mini, illustrating a trade-off between reasoning depth and cost efficiency.

#### Reasoning method cost efficiency

Figure 5 extends our analysis to the full space of model-method combinations. The Pareto frontier, together with the annotated transitions in the figure, reveals two distinct mechanisms for improving medical reasoning, with opposite cost signatures.

**Figure 5:**
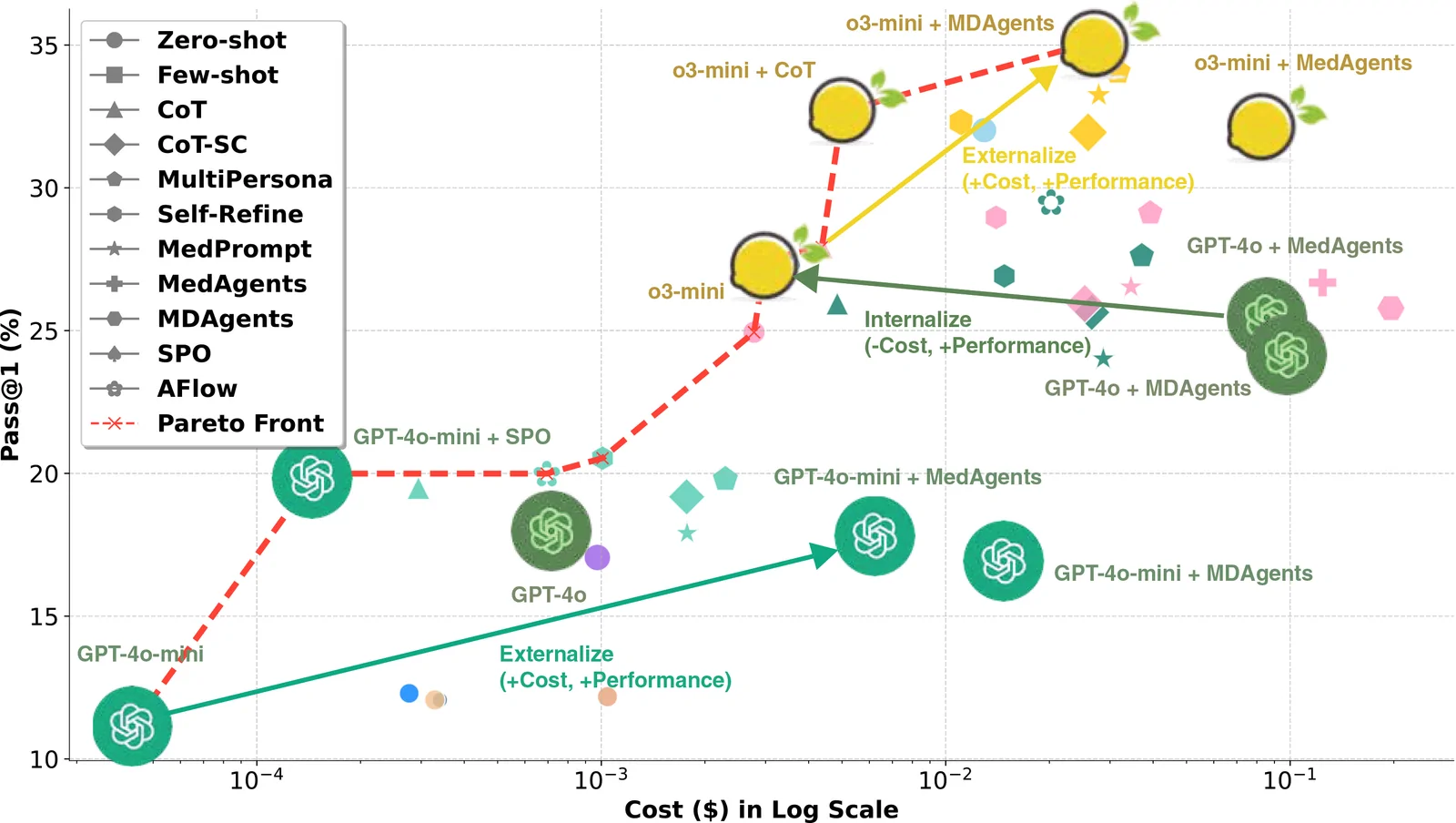
Cost-performance analysis of reasoning methods on MedicalAgentsBench Pass@1 accuracy versus inference cost per sample (log scale). Different markers represent reasoning methods; base-model logo colors follow Figure 1; marker sizes indicate inference time. The red dashed line traces the Pareto frontier of non-dominated configurations. Annotated arrows highlight two distinct mechanisms: externalizing reasoning (adding agent scaffolding to a fixed model) increases both accuracy and cost, while internalizing reasoning (switching to a reasoning model) improves accuracy while reducing inference cost.

##### Externalizing reasoning trades cost for performance

Adding agent scaffolding to a fixed base model consistently improves accuracy but increases inference cost. For instance, moving from bare GPT-4o-mini (11.0%) to GPT-4o-mini + SPO (20.1%) nearly doubles performance at a modest cost increase. However, not all externalizations are equally efficient: GPT-4o-mini + MedAgents (18.3%) costs far more than SPO yet achieves lower accuracy, illustrating that heavier agent scaffolding can yield diminishing or even negative returns on weak base models. The same pattern holds at the top of the frontier: o3-mini zero-shot (27.8%) improves to o3-mini + CoT (32.7%) and then to o3-mini + MDAgents (35.1%), each step adding both accuracy and cost.

##### Internalizing reasoning improves performance while reducing inference cost

Switching from an agent-augmented base model to a bare reasoning model can simultaneously increase accuracy and decrease per-sample inference cost. The most striking example is the transition from the cluster of GPT-4o + agent configurations (25%–30% accuracy at 10×–100× higher inference cost) to bare o3-mini (27.8%): the reasoning model achieves comparable or higher accuracy at an order of magnitude lower inference cost. This reflects the efficiency of internalized reasoning at deployment time, where multi-step inference is compiled into the model’s parameters rather than orchestrated through repeated API calls. We note, however, that this analysis considers only inference costs; the training cost required to develop reasoning capabilities (e.g., reinforcement learning and CoT distillation) is not publicly disclosed by model providers and may be substantial.

These two mechanisms are complementary and together define the Pareto frontier. The optimal strategy traces a path that first externalizes reasoning on cheap models (GPT-4o-mini + SPO), then internalizes by upgrading to a reasoning model (o3-mini), and finally externalizes again by layering agents on the reasoning model (o3-mini + MDAgents). Agent frameworks applied to non-reasoning models (e.g., GPT-4o + MedAgents) fall well below this frontier, as their high inference cost is not justified by commensurate accuracy gains. Practitioners should therefore view internalized reasoning as the primary investment for improving accuracy per dollar, with externalized agent scaffolding as an additive enhancement best applied atop already-strong reasoning models.

## Discussion

The central finding of this study is that internalized reasoning and externalized agent scaffolding are not substitutes but complements. Agents lift base-model accuracy (GPT-4o to GPT-4o + MedAgents), internalized reasoning models do the same at lower inference cost (GPT-4o + agents to o3-mini), and agents on top of internalized reasoning models push accuracy further still (o3-mini to o3-mini + MDAgents: 35.1%). Neither approach subsumes the other. Internalized reasoning models internalize multi-step inference through training-time optimization, while agent frameworks introduce diverse viewpoints and iterative error correction at inference time. The two mechanisms operate on different failure modes, which explains why their gains compound rather than overlap.

The Pareto frontier analysis further clarifies when each approach is most valuable. At the budget-conscious end, lightweight workflow optimization methods such as SPO applied to inexpensive base models (e.g., GPT-4o-mini + SPO) provide the most cost-efficient entry point. At the high-performance end, internalized reasoning models combined with agent methods (o3-mini + CoT and o3-mini + MDAgents) dominate the frontier. Discussion-based frameworks (MedAgents and MDAgents) show their greatest value when paired with internalized reasoning models, where the additional inference cost is justified by meaningful accuracy gains. Our error analysis (Note S1) confirms this mechanistically: multi-agent aggregation amplifies shared biases when agents lack diverse reasoning priors, whereas structured self-critique and role-specialized disagreement can recover from localized errors.

For deployment, these results suggest a simple decision rule. If the budget allows an internalized reasoning model, use one; agents on top will further improve accuracy. If not, lightweight workflow optimization (e.g., SPO on GPT-4o-mini) offers the best accuracy per dollar. In either regime, the choice is not “agents or internalized reasoning models” but “how much of each.” This trade-off is especially salient in healthcare, where privacy requirements and local infrastructure constraints may preclude sending sensitive clinical data to frontier proprietary systems.^20^^,^^52^ In practice, the key question is which combination of internalized reasoning models and externalized workflows best satisfies privacy, compute, latency, and accuracy requirements.

More broadly, MedicalAgentsBench demonstrates the value of decision-oriented benchmarking that jointly evaluates accuracy, cost, and reasoning quality. By filtering for genuinely difficult questions and screening for data contamination, our benchmark exposes meaningful performance differences that standard medical question answering (QA) datasets obscure through ceiling effects.

### Limitations of the study

Our benchmark draws exclusively from multiple-choice QA datasets derived from educational resources, which do not capture the full complexity of clinical practice (e.g., longitudinal patient management, ambiguous presentations, and multi-modal evidence integration). Validating these findings on real-world clinical decision-making tasks remains an important next step.

The 50% accuracy threshold used for difficulty filtering introduces a potential circularity: the hard subset is defined relative to the specific models evaluated. We mitigate this through architectural diversity in the model pool and independent human validation of reasoning depth, but future work should explore task-intrinsic difficulty measures that do not depend on model performance.

Our cost comparisons focus on inference-time expense. For proprietary internalized reasoning models such as o3-mini, the training cost required to produce these reasoning capabilities is not publicly disclosed, making it difficult to compare the full end-to-end economic trade-off between internalizing reasoning during training and externalizing it through agent scaffolding at inference time.

Finally, our evaluation covers a fixed set of nine agentic methods. More sophisticated strategies, such as stepwise verification, heterogeneous model ensembles, and dynamic agent collaboration, remain unexplored and could alter the comparative landscape between internalized and externalized reasoning.

## Methods

### Evaluation tasks and datasets

MedicalAgentsBench is a carefully curated benchmark designed to evaluate complex medical reasoning tasks. We draw from eight established medical datasets, described below.

#### MedQA

MedQA^8^ is a large-scale open-domain medical question-answering dataset collected from professional board exams such as the USMLE. In our benchmark, we use 1,273 English multiple-choice questions that test a broad range of clinical and foundational medical knowledge.

#### PubMedQA

PubMedQA^9^ is a biomedical question-answering dataset constructed from PubMed abstracts to evaluate reasoning over primary research literature. We use 500 expert-annotated yes/no/maybe questions, where each item pairs a research question with its corresponding abstract.

#### MedMCQA

MedMCQA^10^ is a large-scale multiple-choice dataset built from Indian medical entrance exams (AIIMS and NEET PG), covering many subjects and topics. Our benchmark includes 2,816 questions sampled from MedMCQA, focusing on diverse real-world exam-style problems.

#### MedBullets

MedBullets^42^ comprises USMLE Step 2/3-style clinical questions sourced from the MedBullets online learning platform. We use 308 challenging case-based questions, each designed to simulate realistic clinical decision-making scenarios with expert-authored explanations.

#### MedExQA

MedExQA^43^ is a benchmark designed to evaluate medical question answering with a focus on explanation quality across underrepresented specialties. We extract 935 questions spanning audiology, nutrition, occupational therapy, physical therapy, and speech-language pathology, each paired with multiple expert explanations.

#### MedXpertQA

MedXpertQA^44^ is a recent benchmark targeting expert-level medical knowledge and advanced clinical reasoning across multiple specialties and body systems. We include 2,450 text-based questions from MedXpertQA, covering both understanding-oriented and reasoning-intensive scenarios.

#### MMLU

The MMLU benchmark^45^ includes several medically relevant subjects. Following the Med-PaLM setting, we retain only the medical fields clinical_knowledge, professional_medicine, college_medicine, medical_genetics, anatomy, and college_biology, yielding 1,089 questions.

#### MMLU-Pro

MMLU-Pro^46^ is an enhanced, more challenging extension of MMLU that emphasizes reasoning and harder distractors. We include only subjects whose source (src) belongs to the health category, specifically clinical_knowledge, professional_medicine, college_medicine, medical_genetics, nutrition, human_aging, anatomy, and virology, resulting in 818 questions.

These source datasets vary significantly in size (from 308 to 2,816 questions), average token length (18.7–316.1), and number of options (3–10), providing diverse evaluation contexts (Table 2).

### Benchmark construction

As illustrated in Figure 6, we construct MedicalAgentsBench by aggregating questions from multiple medical QA datasets and applying a multi-stage filtering and verification process to ensure difficulty, reasoning depth, and clinical validity. Our hard-set selection process is based on three key criteria.

**Figure 6:**
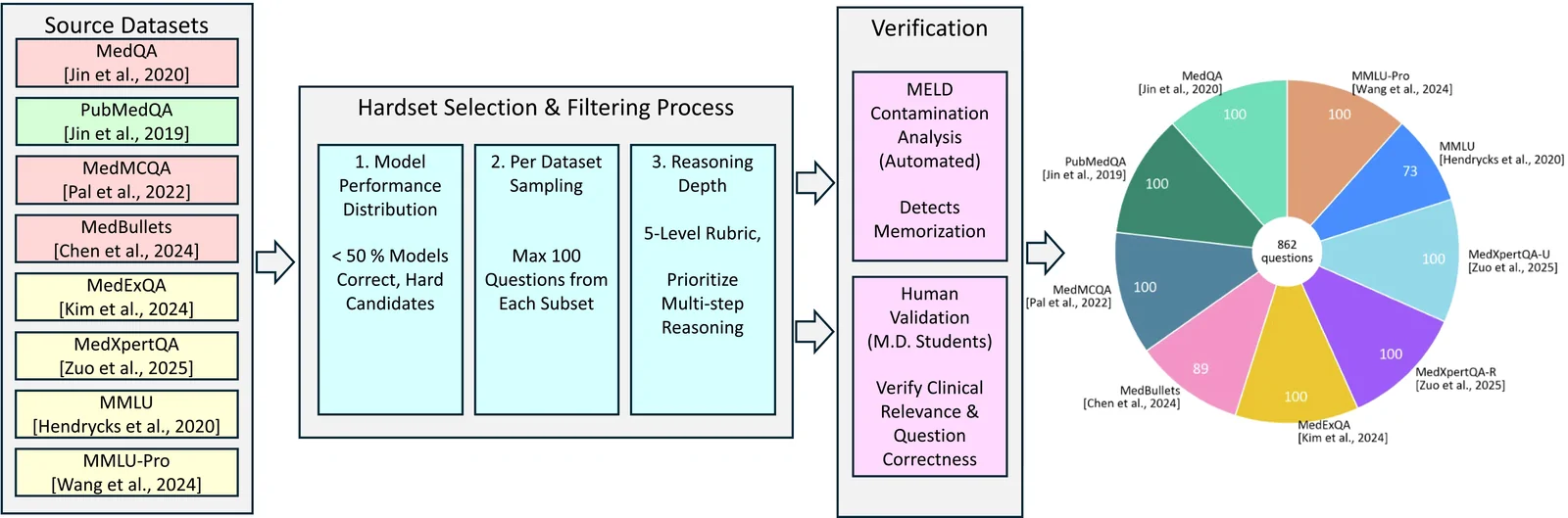
Hard-set construction pipeline for MedicalAgentsBench Questions are aggregated from eight established medical QA datasets and filtered via a three-stage selection process based on model performance, per-dataset sampling, and reasoning depth. The resulting set is verified using automated contamination analysis and human validation by MD students. The final benchmark contains 862 curated hard questions, with per-dataset composition shown on the right.

#### Model performance distribution

As visualized in Figure 7, we analyze the proportion of models that correctly answer each question (*k*/*N* ratio). Questions where less than 50% of models provide correct answers (left of the dashed line in Figure 7) are categorized as hard candidates. This ensures that our benchmark focuses on truly challenging questions that current models struggle with.

**Figure 7:**
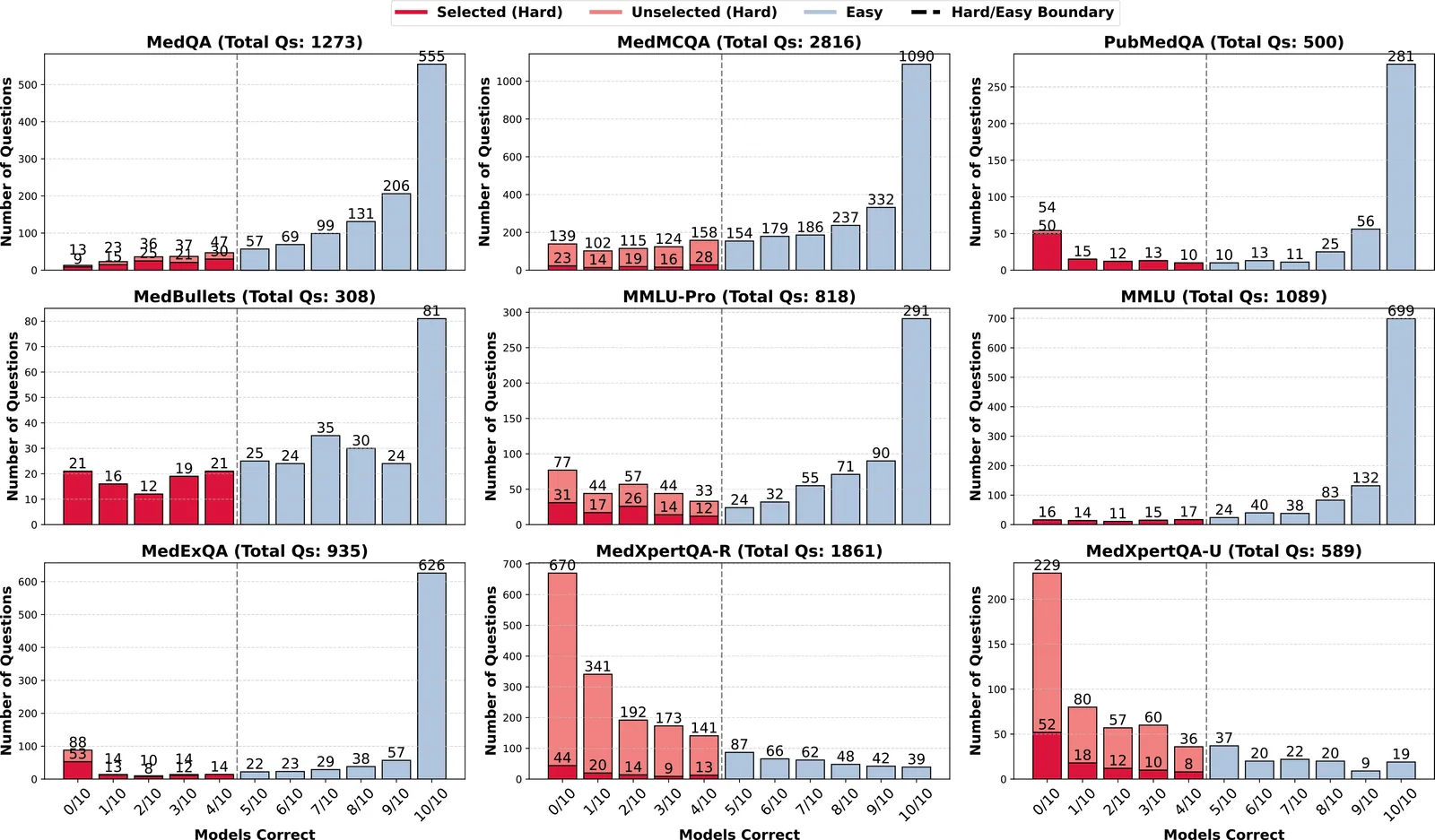
Distribution of model performance across eight medical datasets MedQA, MedMCQA, PubMedQA, MedBullets, MMLU-Pro, MMLU, MedExQA, and MedXpertQA. Each subplot shows the number of questions answered correctly by different proportions of models (*x* axis: *k*/*N*, where *k* is the number of correct models and *N* is the total number of models). Questions are categorized as either hard (left of the dashed line, <50% of models correct) or easy (right of the dashed line, ≥50% of models correct), with selected questions highlighted in darker shades. The total question count for each dataset is indicated in the subplot titles.

#### Dataset sampling strategy

We carefully balanced our benchmark to ensure comprehensive coverage across various source datasets. Our hard subset includes exactly 100 questions each from MedQA, PubMedQA, MedMCQA, MMLU-Pro, and MedExQA. From MedXpertQA, we incorporated both its reasoning (MedXpertQA-R) and understanding (MedXpertQA-U) subsets (100 questions each), which were annotated in the original paper to distinguish between questions that require complex clinical reasoning and those primarily testing medical knowledge. Additionally, we included 89 questions from MedBullets and 73 questions from MMLU. This sampling distribution maintains proportional representation across the source datasets while ensuring sufficient sample size to evaluate model performance across different medical question types and their associated reasoning requirements.

#### Reasoning depth

We prioritize questions that require multi-step reasoning processes, where solving the problem involves a sequence of logical deductions or inferences rather than simple fact retrieval. This type of reasoning is typically demonstrated in long CoT approaches, where models must work through several intermediate steps to reach a conclusion. To systematically assess reasoning depth, we developed a five-level classification rubric and engaged medical professionals to annotate each question (see the human validation protocol below for the complete rubric and validation details). The need for such complex reasoning is evidenced by the significant performance gap between base models and agent-based approaches. As shown in Table 3, while models achieve high accuracy on the full set (e.g., GPT-4o: 88.2% on MedQA), their performance drops significantly on our hard subset (e.g., GPT-4o: 32.2% on MedQA-Hard), confirming that these selected questions demand deeper analytical processing and medical reasoning capabilities.

In summary, we construct MedicalAgentsBench through a multi-stage pipeline designed to ensure question difficulty, diversity, and evaluation validity. Specifically, we first evaluate each candidate question across multiple model architectures (as shown in Table 3) to ensure architecture-independent difficulty. Second, we apply a difficulty-aware dataset sampling strategy to balance coverage across source benchmarks. Third, we incorporate human annotations to verify that selected questions require multi-step medical reasoning rather than surface-level recall. Finally, we conduct a data contamination analysis using MELD^51^ to identify questions that may have been memorized during model training. This analysis involves splitting each test item (including the question text but excluding answer options) into two halves, providing models with only the first half, and measuring the similarity between their generated continuations and the original second half. By filtering out potentially memorized items, this step safeguards the fairness and validity of the benchmark for evaluating genuine medical reasoning capabilities. As shown in Figure 3, the hard subset selected for our MedicalAgentsBench benchmark consistently demonstrates lower similarity scores (20%–40%) than the original datasets, suggesting our filtering process effectively selects questions that test genuine reasoning rather than memorization. Complete details of our MELD methodology and its limitations are discussed in the data contamination analysis in the results. Finally, four medical professionals (MD students) review the final question set to verify clinical relevance and reasoning complexity by using the validation protocol described below. The resulting MedicalAgentsBench benchmark contains 862 questions with an average token length of 147.4.

### Human validation protocol

To ensure the quality and clinical validity of benchmark questions, we conducted a human validation study with four medical students in their clinical years with formal clinical training as annotators. The validation process served two primary objectives: assessing the level of reasoning depth required to answer each question and verifying the correctness and clinical relevance of the questions. Each annotator received standardized training materials and participated in a calibration session, during which they jointly reviewed and discussed 10 representative sample questions to ensure consistent interpretation and application of the evaluation criteria. Following calibration, annotators independently reviewed assigned question subsets, with overlapping samples included to enable estimation of inter-annotator agreement.

#### Reasoning depth assessment

For reasoning depth assessment, we developed a systematic rubric that defines reasoning depth as the minimum number of distinct cognitive inference steps required to arrive at the correct answer. Each step corresponds to a discrete inference, calculation, or clinical judgment that builds upon previously derived information. Table S1 summarizes the five-level reasoning depth classification system and provides representative clinical examples for each level. Annotators independently reviewed each question, documented the required reasoning steps, and assigned depth scores according to this rubric. To enable reliable estimation of inter-annotator agreement, we sampled 20 questions from each dataset (10 from the original dataset and 10 from the curated hard subset) for comparative annotation by multiple reviewers.

Inter-annotator reliability was quantified using Fleiss’ *κ*, which measures agreement beyond chance among multiple raters. Specifically, for each item, we compute the proportion of rater pairs that assign the same category and average this quantity across items to obtain the observed agreement $\bar{P}$. The expected agreement ${\bar{P}}_{e}$ is computed from the squared category frequencies averaged across all ratings. Fleiss’ *κ* is then defined as

$$\kappa =\frac{\bar{P}-{\bar{P}}_{e}}{1-{\bar{P}}_{e}}\text{.}$$

Across all datasets, the resulting inter-annotator agreement is *κ* = 0.62 (Table S3), which is commonly considered substantial agreement for complex cognitive annotation tasks.

#### Question validity assessment

Concurrent with reasoning depth assessment, annotators evaluated question validity using strict exclusion criteria. Questions were flagged for removal if they contained wrong answers contradicting current medical guidelines, lacked sufficient clinical context for definitive answers, or tested outdated or clinically irrelevant knowledge that could be reasonably interpreted multiple ways. Table S2 summarizes our question exclusion criteria and the review process.

Our quality control protocol implemented several measures to ensure annotation consistency. Following the initial calibration phase, any question flagged by at least one annotator underwent group review. Questions were removed from the final dataset if two or more annotators agreed on the validity of the concern, with all exclusions documented with a specific rationale. Of the initial candidate questions, 21 questions were removed due to validity concerns: 5 for wrong answers, 5 for poor clinical context, and 11 for clinical irrelevance.

### LLMs

We conduct a comprehensive evaluation of both base models and agentic reasoning methods across our MedicalAgentsBench benchmark. Our experiments follow a standardized protocol to ensure fair comparison, with consistent prompting strategies and evaluation metrics across all models and methods.

#### Representative LLMs and their versions

For base-model comparison, we evaluate both closed-source models (GPT-4o, GPT-4o-mini, Claude-3.5-Sonnet, Claude-3.5-Haiku, o1-mini, and o3-mini) and open-source alternatives (DeepSeek-V3, DeepSeek-R1, Llama-3.3-70B, and QwQ-32B). Specific model versions were accessed between January and February 2025. For closed-source models via OpenAI and Anthropic APIs, we used gpt-4o-2024-08-06, gpt-4o-mini-2024-07-18, claude-3-5-sonnet-20241022, claude-3-5-haiku-20241022, o1-mini-2024-09-12, and o3-mini-2025-01-31. For open-source models hosted on Together AI (https://www.together.ai/), we used deepseek-ai/DeepSeek-V3, deepseek-ai/DeepSeek-R1, meta-llama/Llama-3.3-70B-Instruct-Turbo, and Qwen/QwQ-32B-Preview. Note that Together AI has since updated DeepSeek-V3 to V3.1 and retired QwQ-32B-Preview.

#### Reasoning methods

Additionally, we evaluate 9 distinct agentic reasoning approaches spanning three categories. The first category includes baseline prompting methods such as CoT and SC. The second encompasses advanced prompting techniques: Multi-Persona, Self-Refine, and MedPrompt. The third category covers agent-based frameworks, including medical-specific collaboration frameworks (MedAgents and MDAgents, which we reimplemented to ensure accurate parsing of multiple-choice responses) and search-based agent methods (SPO and AFlow) with search strategies consistent with the original setting. Because agentic pipelines require extensive multi-step reasoning, evaluating them on all existing medical benchmarks would be computationally prohibitive. Therefore, Table 1 reports agentic results solely on MedicalAgentsBench, while Table 2 provides cross-benchmark comparisons for single-call model evaluations. Our implementations of these baseline methods and evaluation framework are publicly available at https://github.com/gersteinlab/MedicalAgentsBench/.

Our data contamination analysis (Figure 3) revealed that OpenAI models demonstrate consistently lower memorization metrics across all datasets than other model families. This finding guided our decision to use GPT-4o, GPT-4o-mini, and o3-mini as primary models for method comparison to minimize performance advantages stemming from potential training data contamination.

#### Standardized evaluation

All experiments utilize identical prompt templates and evaluation protocols to ensure a fair comparison. We implement a standardized two-round inference protocol per query for agent-based methods requiring multiple inference rounds (e.g., MedAgents). Multi-agent approaches (e.g., MDAgents) consistently employ three distinct agent roles for all evaluations. This standardization mitigates confounding factors arising from implementation variations, allowing us to more accurately attribute performance differences to the methods themselves rather than to differences in their implementations.

### Cost analysis

To analyze cost-performance trade-offs, we followed a standardized evaluation protocol. Understanding these trade-offs is particularly important given the significant computational resources required by advanced reasoning models, which often come with substantially higher inference costs and longer processing times than traditional models. Similarly, multi-agent frameworks require multiple rounds of API calls for agent interactions, further increasing computational costs and inference time. In this analysis, we focus exclusively on inference-time cost and latency, as training costs are highly model dependent, often proprietary, and difficult to attribute fairly on a per-task or per-sample basis.

For API-based commercial models (OpenAI and Claude), we calculated costs using their published pricing rates based on total token usage (input + output). For open-source models (such as DeepSeek, Llama, and QwQ), we estimated costs based on Together AI’s hosting rates (https://www.together.ai/), a platform that provides API access to open-source models. This approach enables fair cost comparisons between proprietary and open-source alternatives across different deployment scenarios. The total cost of experimentation was $226.17. We measured inference time as wall-clock time per sample, including prompt construction and model inference, across agent-based methods that included their complete interaction cycles.

## Resource availability

### Lead contact

Requests for further information and resources should be directed to and will be fulfilled by the lead contact, Mark Gerstein (pi@gersteinlab.org).

### Materials availability

This study did not generate new physical materials.

### Data and code availability

The benchmark dataset and all original code reported in this study are publicly available. The benchmark dataset is deposited at Zenodo^38^: https://doi.org/10.5281/zenodo.18944609 and Hugging Face: https://huggingface.co/datasets/super-dainiu/MedicalAgentsBench. The code is deposited at Zenodo^39^: https://doi.org/10.5281/zenodo.18944554 and GitHub: https://github.com/gersteinlab/MedicalAgentsBench. Any additional information required to reanalyze the data reported in this paper is available from the lead contact upon request.

## Acknowledgments

We thank the anonymous reviewers for their constructive feedback. This work was supported by the ALW Professorship Funds and the 10.13039/100000002National Institutes of Health grant R01DA063148.

## Author contributions

Y.S., X.T., and J.S. conceptualized the study and methodology. Y.S. collected and curated data, developed software, conducted experiments, and created figures. J.S. curated data. J.C. and Y.L. created the figures. J.Z. and J.X. conducted the baseline experiments. Y.S., X.T., J.C., and J.S. wrote the manuscript. F.W., Y.Z., C.W., W.S., and A.C. reviewed and edited the manuscript. M.G. and X.T. supervised the project.

## Declaration of interests

The authors declare no competing interests.

## Declaration of generative AI and AI-assisted technologies in the writing process

During the preparation of this work, the authors used Claude (Anthropic) to assist with manuscript formatting, editing, and bibliography management. After using this tool/service, the authors reviewed and edited the content as needed and take full responsibility for the content of the published article.

## Supplemental information

Document S1. Tables S1–S8 and Note S1

Document S2. Article plus supplemental information

## References

[1] Singhal K., Tu T., Gottweis J., Sayres R., Wulczyn E., Amin M., Hou L., Clark K., Pfohl S.R., Cole-Lewis H. (2025). Toward expert-level medical question answering with large language models. Nat. Med..

[2] Jin Q., Yuan Z., Xiong G., Yu Q., Ying H., Tan C., Chen M., Huang S., Liu X., Yu S. (2022). Biomedical question answering: a survey of approaches and challenges. ACM Comput. Surv..

[3] Chen Q., Hu Y., Peng X., Xie Q., Jin Q., Gilson A., Singer M.B., Ai X., Lai P.T., Wang Z. (2025). Benchmarking large language models for biomedical natural language processing applications and recommendations. Nat. Commun..

[4] Liu F., Zhou H., Gu B., Zou X., Huang J., Wu J., Li Y., Chen S.S., Hua Y., Zhou P. (2025). Application of large language models in medicine. Nat. Rev. Bioeng..

[5] Gao S., Fang A., Huang Y., Giunchiglia V., Noori A., Schwarz J.R., Ektefaie Y., Kondic J., Zitnik M. (2024). Empowering biomedical discovery with AI agents. Cell.

[6] Fan Z., Wei L., Tang J., Chen W., Siyuan W., Wei Z., Huang F. (2025). Proceedings of the 31st International Conference on Computational Linguistics.

[7] Shi W., Xu R., Zhuang Y., Yu Y., Sun H., Wu H., Yang C., Wang M.D. (2024). Proceedings of the 2024 Conference on Empirical Methods in Natural Language Processing.

[8] Jin D., Pan E., Oufattole N., Weng W.H., Fang H., Szolovits P. (2021). What disease does this patient have? A large-scale open domain question answering dataset from medical exams. Appl. Sci..

[9] Jin Q., Dhingra B., Liu Z., Cohen W.W., Lu X. (2019). Proceedings of the 2019 Conference on Empirical Methods in Natural Language Processing.

[10] Pal A., Umapathi L.K., Sankarasubbu M. (2022). Conference on Health, Inference, and Learning.

[11] Pampari A., Raghavan P., Liang J., Peng J. (2018). Proceedings of the 2018 Conference on Empirical Methods in Natural Language Processing.

[12] Abacha A.B., M’rabet Y., Zhang Y., Shivade C., Langlotz C., Demner-Fushman D. (2021). Proceedings of the 20th Workshop on Biomedical Language Processing.

[13] He X., Zhang Y., Mou L., Xing E., Xie P. (2020). PathVQA: 30000+ questions for medical visual question answering. arXiv.

[14] Soni S., Gudala M., Pajouhi A., Roberts K. (2022). Proceedings of the 13th Language Resources and Evaluation Conference.

[15] Bae S., Kyung D., Ryu J., Cho E., Lee G., Kweon S., Oh J., Ji L., Chang E., Kim T. (2023). EHRXQA: A multi-modal question answering dataset for electronic health records with chest x-ray images. Adv. Neural Inf. Process. Syst..

[16] Vilares D., Gómez-Rodríguez C. (2019). Proceedings of the 57th Annual Meeting of the Association for Computational Linguistics.

[17] Hertzberg N., Lokrantz A. (2024). Proceedings of the 2024 Joint International Conference on Computational Linguistics, Language Resources and Evaluation.

[18] Olatunji T., Nimo C., Owodunni A., Abdullahi T., Ayodele E., Sanni M., Aka C., Omofoye F., Yuehgoh F., Faniran T. (2025). Proceedings of the 63rd Annual Meeting of the Association for Computational Linguistics.

[19] Singhal K., Azizi S., Tu T., Mahdavi S.S., Wei J., Chung H.W., Scales N., Tanwani A., Cole-Lewis H., Pfohl S. (2023). Large language models encode clinical knowledge. Nature.

[20] Thirunavukarasu A.J., Ting D.S.J., Elangovan K., Gutierrez L., Tan T.F., Ting D.S.W. (2023). Large language models in medicine. Nat. Med..

[21] Liévin V., Hother C.E., Motzfeldt A.G., Winther O. (2024). Can large language models reason about medical questions?. Patterns.

[22] Gilson A., Safranek C.W., Huang T., Socrates V., Chi L., Taylor R.A., Chartash D. (2023). How does ChatGPT perform on the United States Medical Licensing Examination (USMLE)? the implications of large language models for medical education and knowledge assessment. JMIR Med. Educ..

[23] Achiam J., Adler S., Agarwal S., Ahmad L., Akkaya I., Aleman F.L., Almeida D., Altenschmidt J., Altman S., Anadkat S. (2023). GPT-4 technical report. arXiv.

[24] Han T., Adams L.C., Papaioannou J.M., Grundmann P., Oberhauser T., Löser A., Truhn D., Bressem K.K. (2023). MedAlpaca–an open-source collection of medical conversational AI models and training data. arXiv.

[25] Wu C., Lin W., Zhang X., Zhang Y., Xie W., Wang Y. (2024). PMC-LLaMA: toward building open-source language models for medicine. J. Am. Med. Inform. Assoc..

[26] Chen Z., Cano A.H., Romanou A., Bonnet A., Matoba K., Salvi F., Pagliardini M., Fan S., Köpf A., Mohtashami A. (2023). Meditron-70b: Scaling medical pretraining for large language models. arXiv.

[27] Liu J., Wang Y., Du J., Zhou J.T., Liu Z. (2024). Proceedings of the 2024 Conference on Empirical Methods in Natural Language Processing.

[28] Jin Q., Kim W., Chen Q., Comeau D.C., Yeganova L., Wilbur W.J., Lu Z. (2023). MedCPT: Contrastive pre-trained transformers with large-scale pubmed search logs for zero-shot biomedical information retrieval. Bioinformatics.

[29] Jeong M., Sohn J., Sung M., Kang J. (2024). Improving medical reasoning through retrieval and self-reflection with retrieval-augmented LLMs. Bioinformatics.

[30] Xiong G., Jin Q., Lu Z., Zhang A. (2024). Findings of the Association for Computational Linguistics.

[31] Tang X., Zou A., Zhang Z., Li Z., Zhao Y., Zhang X., Cohan A., Gerstein M. (2024). Findings of the Association for Computational Linguistics ACL 2024.

[32] Kim Y., Park C., Jeong H., Chan Y.S., Xu X., McDuff D., Lee H., Ghassemi M., Breazeal C., Park H.W. (2024). MDAgents: An adaptive collaboration of llms in medical decision making. Adv. Neural Inf. Process. Syst..

[33] Wei J., Wang X., Schuurmans D., Bosma M., Xia F., Chi E., Le Q.V., Zhou D. (2022). Chain-of-thought prompting elicits reasoning in large language models. Adv. Neural Inf. Process. Syst..

[34] Wang X., Wei J., Schuurmans D., Le Q., Chi E., Narang S., Chowdhery A., Zhou D. (2023). International Conference on Learning Representations.

[35] Nori H., Lee Y.T., Zhang S., Carignan D., Edgar R., Fusi N., King N., Larson J., Li Y., Liu W. (2023). Can generalist foundation models outcompete special-purpose tuning? Case study in medicine. arXiv.

[36] Zhang J., Xiang J., Yu Z., Teng F., Chen X., Chen J., Zhuge M., Cheng X., Hong S., Wang J. (2025). International Conference on Learning Representations.

[37] Xiang J., Zhang J., Yu Z., Teng F., Tu J., Liang X., Hong S., Wu C., Luo Y. (2025). Findings of the Association for Computational Linguistics: EMNLP 2025.

[38] Shao Y., Tang X., Sohn J., Chen J., Liao Y., Zhang J., Xiang J., Wu F., Zhao Y., Wu C., Shi W. (2026). MedicalAgentsBench: A benchmark of 862 complex clinical questions (dataset). Zenodo.

[39] Shao Y., Tang X., Sohn J., Chen J., Liao Y., Zhang J., Xiang J., Wu F., Zhao Y., Wu C., Shi W. (2026). MedicalAgentsBench: Evaluation frame- work and baseline implementations (code). Zenodo.

[40] Guo D., Yang D., Zhang H., Song J., Wang P., Zhu Q., Xu R., Zhang R., Ma S., Bi X. (2025). DeepSeek-R1 incentivizes reasoning in LLMs through reinforcement learning. Nature.

[41] Raschka S. (2025). Understanding reasoning LLMs: Methods and strategies for building and refining reasoning models. Ahead of AI.

[42] Chen H., Fang Z., Singla Y., Dredze M. (2025). Proceedings of the 2025 Conference of the Nations of the Americas Chapter of the Association for Computational Linguistics.

[43] Kim Y., Wu J., Abdulle Y., Wu H. (2024). Processing BioNLP Workshop.

[44] Zuo Y., Qu S., Li Y., Chen Z., Zhu X., Hua E., Zhang K., Ding N., Zhou B. (2025). Proceedings of the 42nd International Conference on Machine Learning vol. 267 of Proceedings of Machine Learning Research.

[45] Hendrycks D., Burns C., Basart S., Zou A., Mazeika M., Song D., Steinhardt J. (2021). International Conference on Learning Representations.

[46] Wang Y., Ma X., Zhang G., Ni Y., Chandra A., Guo S., Ren W., Arulraj A., He X., Jiang Z. (2024). MMLU-Pro: A more robust and challenging multi-task language understanding benchmark. Adv. Neural Inf. Process. Syst..

[47] Bedi S., Cui H., Fuentes M., Unell A., Wornow M., Banda J.M., Kotecha N., Keyes T., Mai Y., Oez M. (2026). Holistic evaluation of large language models for medical tasks with MedHELM. Nat. Med..

[48] Jiang Y., Black K.C., Geng G., Park D., Zou J., Ng A.Y., Chen J.H. (2025). MedAgentBench: A virtual EHR environment to benchmark medical LLM agents. NEJM AI.

[49] Zhu Y., He Z., Hu H., Zheng X., Zhang X., Wang Z., Gao J., Ma L., Yu L. (2025). MedAgentBoard: Benchmarking multi-agent collaboration with conventional methods for diverse medical tasks. Adv. Neural Inf. Process. Syst..

[50] Xu R., Zhuang Y., Zhong Y., Yu Y., Wang Z., Tang X., Wu H., Wang M.D., Ruan P., Yang D. (2026). International Conference on Learning Representations.

[51] Nori H., King N., McKinney S.M., Carignan D., Horvitz E. (2023). Capabilities of GPT-4 on medical challenge problems. arXiv.

[52] Peng C., Yang X., Chen A., Smith K.E., PourNejatian N., Costa A.B., Martin C., Flores M.G., Zhang Y., Magoc T. (2023). A study of generative large language model for medical research and healthcare. NPJ Digit. Med..

