# MedicalAgentsBench for complex medical reasoning: Comparing internalized reasoning models versus externalized agent-based frameworks

**DOI:** 10.1016/j.patter.2026.101658

**Published:** 2026-08-25

**Authors:** Yanjun Shao, Xiangru Tang, Jiwoong Sohn, Jiapeng Chen, Yuxuan Liao, Jiayi Zhang, Jinyu Xiang, Fang Wu, Yilun Zhao, Chenglin Wu, Wenqi Shi, Arman Cohan, Mark Gerstein

## Main text

(Patterns *7*, 101610; October 9, 2026)

In the originally published version of the article, the author Yuxuan Liao was incorrectly placed within the author list. This error occurred during production due to an oversight by the production staff. The author list appears correctly here and has been corrected online with the original article. Cell Press apologizes to the authors and readers for the inconvenience.

